# Automatic identification of inertial sensor placement on human body segments during walking

**DOI:** 10.1186/1743-0003-10-31

**Published:** 2013-03-21

**Authors:** Dirk Weenk, Bert-Jan F van Beijnum, Chris TM Baten, Hermie J Hermens, Peter H Veltink

**Affiliations:** 1 MIRA Institute for Biomedical Technology and Technical Medicine, Biomedical Signals and Systems, University of Twente, Enschede, The Netherlands; 2 CTIT Centre for Telematics and Information Technology, Biomedical Signals and Systems, University of Twente, Enschede, The Netherlands; 3 Roessingh Research and Development, , Enschede, The Netherlands

**Keywords:** Inertial sensors, Feature extraction, Classification, Decision trees, Weka

## Abstract

**Background:**

Current inertial motion capture systems are rarely used in biomedical applications. The attachment and connection of the sensors with cables is often a complex and time consuming task. Moreover, it is prone to errors, because each sensor has to be attached to a predefined body segment. By using wireless inertial sensors and automatic identification of their positions on the human body, the complexity of the set-up can be reduced and incorrect attachments are avoided.

We present a novel method for the automatic identification of inertial sensors on human body segments during walking. This method allows the user to place (wireless) inertial sensors on arbitrary body segments. Next, the user walks for just a few seconds and the segment to which each sensor is attached is identified automatically.

**Methods:**

Walking data was recorded from ten healthy subjects using an Xsens MVN Biomech system with full-body configuration (17 inertial sensors). Subjects were asked to walk for about 6 seconds at normal walking speed (about 5 km/h). After rotating the sensor data to a global coordinate frame with *x*-axis in walking direction, *y*-axis pointing left and *z*-axis vertical, RMS, mean, and correlation coefficient features were extracted from *x*-, *y*- and *z*-components and magnitudes of the accelerations, angular velocities and angular accelerations. As a classifier, a decision tree based on the C4.5 algorithm was developed using Weka (Waikato Environment for Knowledge Analysis).

**Results and conclusions:**

After testing the algorithm with 10-fold cross-validation using 31 walking trials (involving 527 sensors), 514 sensors were correctly classified (97.5%). When a decision tree for a lower body plus trunk configuration (8 inertial sensors) was trained and tested using 10-fold cross-validation, 100% of the sensors were correctly identified. This decision tree was also tested on walking trials of 7 patients (17 walking trials) after anterior cruciate ligament reconstruction, which also resulted in 100% correct identification, thus illustrating the robustness of the method.

## Background

Conventional human motion capture systems make use of cameras and are therefore bounded to a restricted area. This is one of the reasons why over the last few years, inertial sensors (accelerometers and gyroscopes) in combination with magnetic sensors were demonstrated to be a suitable ambulatory alternative. Although accurate 6 degrees of freedom information is available [1], these inertial sensor systems are rarely used in biomedical applications, for example rehabilitation and sports training. This unpopularity could be related to the set-up of the systems. The attachment and connection of the sensors with cables is often a complex and time consuming task. Moreover, it is prone to errors, because each sensor has to be attached to a predefined body segment. Despite the fact that the set-up time for inertial systems is significantly lower (≤ 15 minutes for an Xsens MVN Biomech system [2]) than for optical systems [3], it is still a significant amount of time.

However, with decreasing sensor sizes and upcoming wireless inertial sensor technology, the inertial sensors can be attached to the body more easily and quickly, for example using Velcro ^*â“‡*^ straps [4] or even plasters [5]. If it were not necessary to attach each sensor to a predefined segment and if the wired inertial sensors were to be replaced by wireless sensors, the system could be easier to use and both the set-up time and the number of attachment errors could be reduced.

A number of studies on localization of body worn sensors have been conducted previously. Kunze *et al.*[6,7] used accelerometer data from 5 inertial sensors combined with various classification algorithms for on-body device localization, resulting in an accuracy of up to 100% for walking and up to 82% for arbitrary activities (92% when using 4 sensors). Amini *et al.*[8] used accelerometer data of 10 sensors combined with an SVM (support vector machine) classifier to determine the on-body sensor locations. An accuracy of 89% was achieved. Despite their promising results, several important questions remain. For example, the robustness of these algorithms was not tested on patients with movement disorders. Additionally, a limited number of sensors was used and no method for identifying left and right limbs was presented.

In order for ambulatory movement analysis systems to become generally accepted in biomedical applications, it is essential that the systems become easier to use. By making the systems plug and play, they can be used without having prior knowledge about technical details of the system and they become robust against incorrect sensor placement. This way clinicians or even the patients themselves can attach the sensors, even if they are at home.

In this paper, a method for automatic identification of body segments to which (wireless) inertial sensors are attached is presented. This method allows the user to place inertial sensors on arbitrary segments of the human body, in a full body- or a lower body plus trunk configuration (17 or 8 inertial sensors respectively). Next, the user walks for just a few seconds and the body segment to which each sensor is attached is identified automatically, based on acceleration and angular velocity data. Walking data was used, because it is often used for motion analysis during rehabilitation. In addition to healthy subjects, the method is tested on a group of 7 patients after anterior cruciate ligament (ACL) reconstruction, using a lower body plus trunk configuration.

## Methods

### Measurements

From 11 healthy subjects (2 female and 9 male students, all between 20-30 years old), 35 walking trials were recorded using an Xsens MVN Biomech system [2] with full body configuration, that is, 17 inertial sensors were placed on 17 different body segments: pelvis, sternum, head, right shoulder, right upper arm, right forearm, right hand, left shoulder, left upper arm, left forearm, left hand, right upper leg, right lower leg, right foot, left upper leg, left lower leg and left foot [9]. The subjects, wearing their own daily shoes (no high heels), were asked to stand still for a few seconds and then to start walking at normal speed (about 5 km/h). Because the data was obtained from different previous studies, the number of trials per subject varied from one to four trials. Also the length of the trials varied. From each trial the first 3 walking cycles (about 6 seconds) were used, which was the minimum available number for several trials. Walking cycles were obtained using peak detection of the summation of magnitudes of accelerations and angular velocities of all sensors ($\overset{n}{\underset{i=1}{\sum }}(∥a_{i}∥+∥\omega_{i}∥)$, where *n* is the number of sensors). One subject (4 trials) showed little to no arm movement during walking and was excluded from the analysis, hence 31 walking trials were used for developing our identification algorithm.

Inertial sensor data – that is, 3D measured acceleration (***s***^*s*^) and 3D angular velocity (***ω***^*s*^), both expressed in sensor coordinate frame – recorded with a sampling frequency of 120 Hz was saved in MVN file format, converted to XML and loaded into MATLAB ^*â“‡*^ for further analysis.

Besides the full-body configuration a subset of this configuration was analyzed. This lower body plus trunk configuration contained 8 inertial sensors placed on 8 different body segments: pelvis, sternum, upper legs, lower legs and feet. In addition to lower body information, the sternum sensor provides important information about the movement of the trunk. This can be useful in applications where balance needs to be assessed.

In order to test the robustness of the algorithm, 17 walking trials of 7 patients (1 female, 6 male, age 28 ±8.35) after anterior cruciate ligament (ACL) reconstruction were used. These trials were recorded using an Xbus Kit (Xsens Technologies B.V. [2]) during a study of Baten *et al.*[10]. In their study 7 patients were measured four times during the rehabilitation process, with an interval of one month. To test the robustness of our identification algorithm, the first measurements – approximately 5 weeks after the ACL reconstruction, where walking asymmetry was largest – were used. No medical ethical approval was required under Dutch regulations, given the materials and methods used. The research was in full compliance with the “Declaration of Helsinki” and written informed consent was obtained from all patients for publication of the results.

### Preprocessing

**Figure 1:**
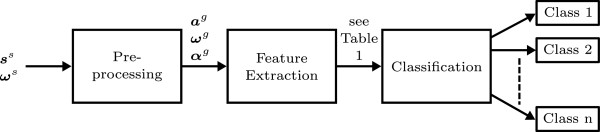
**The three steps used for identifying the inertial sensors.** Inputs are the measured 3D acceleration (***s***^*s*^) and angular velocity (***ω***^*s*^), both expressed in sensor coordinate frame. Outputs of the identification process are the classes, in this case the body segments to which the inertial sensors are attached.

Identification of the inertial sensors was split into three steps: preprocessing, feature extraction and classification (Figure 1). To be able to compare the sensors between different body segments and different subjects, the accelerations and angular velocities were pre-processed; that is, the gravitational accelerations were subtracted from the accelerometer outputs and the 3D sensor signals were all transformed to the global coordinate frame *ψ*_*g*_ with the *z*-axis pointing up, the *x*-axis in the walking direction and the *y*-axis pointing left.

To transform the 3D accelerations and angular velocities from sensor coordinate frame *ψ*_*s*_ to global coordinate frame *ψ*_*g*_, the orientation of the inertial sensor – with respect to the global coordinate frame – had to be estimated. For this purpose, first the inclination of the sensors was estimated when the subjects were standing still, by using the accelerometers that measure the gravitational acceleration under this condition. When the subjects were walking, the change of orientation of the sensors was estimated using the gyroscopes by integrating the angular velocities. The following differential equation was solved to integrate the angular velocities to angles [11]:

(1) $${\dot{R}}_{s}^{g^{\prime }}=R_{s}^{g^{\prime }}{\tilde{\omega }}^{s}$$

where the 3D rotation matrix $R_{s}^{g^{\prime }}$ represents the change of coordinates from *ψ*_*s*_ to a frame $\psi_{g^{\prime }}$ with all vertical axes aligned, but with the heading in the original (unchanged) direction. ${\tilde{\omega }}^{s}$ is a skew-symmetric matrix consisting of the components of the angular velocity vector expressed in *ψ*_*s*_:

(2) $${\tilde{\omega }}^{s}=\left(\begin{array}{ccc} 0 & -\omega_{z} & \omega_{y}\\ \omega_{z} & 0 & -\omega_{x}\\ -\omega_{y} & \omega_{x} & 0 \end{array}\right)$$

where the indices ()^*s*^ are omitted for readability (see also [11]). For the 3D sensor acceleration in frame $\psi_{g^{\prime }}$, denoted $a^{g^{\prime }}(t)$, the following equation holds:

(3) $$a^{g^{\prime }}(t)=R_{s}^{g^{\prime }}(t)s^{s}(t)+g^{g^{\prime }}$$

where ***s***^*s*^(*t*) is the measured acceleration and $g^{g^{\prime }}$ is the gravitational acceleration expressed in $\psi_{g^{\prime }}$ (assumed to be constant and known), which was subsequently subtracted from the *z*-component of the 3D sensor acceleration. The rotation matrix $R_{s}^{g^{\prime }}(t)$ was also used to express ***ω***^*s*^ in $\psi_{g^{\prime }}$:

(4) $$\omega^{g^{\prime }}(t)=R_{s}^{g^{\prime }}(t)\omega^{s}(t)$$

After aligning the vertical axes, the heading was aligned by aligning the positive $x^{g^{\prime }}$-axis with the walking direction, which was obtained by integrating the acceleration in frame $\psi_{g^{\prime }}$ – yielding the velocity $v^{g^{\prime }}$ – using trapezoidal numerical integration. From $v^{g^{\prime }}$, the *x* and *y* components were used to obtain the angle (in the horizontal plane) with the positive *x*-axis ($x^{g^{\prime }}$). Drawback of this method is the drift caused by integrating noise and sensor bias. The effect of this integration drift on the estimation of the walking direction was reduced by using the mean of the velocity of the first full walking cycle to estimate the walking direction, assuming that this gave a good estimate of the walking direction of the complete walking trial.

The angle *θ* (in the horizontal plane) between $x^{g^{\prime }}$ and the velocity vector $v^{g^{\prime }}$ was obtained using:

(5) $$\theta =\arccos\left(\frac{x^{g^{\prime }}\cdot v^{g^{\prime }}}{∥x^{g^{\prime }}∥∥v^{g^{\prime }}∥}\right)$$

This angle was then used to obtain the rotation matrix:

(6) $$R_{g^{\prime }}^{g}(\theta )=\left(\begin{array}{ccc} \cos\theta & \;-\sin\theta & \;0\\ \sin\theta & \;\cos\theta & \;0\\ 0 & \;0 & \;1 \end{array}\;\right)$$

which was used (as in (4)) to rotate the accelerations ($a^{g^{\prime }}$) and angular velocities ($\omega^{g^{\prime }}$) of all the sensors to global coordinate frame *ψ*_*g*_, with *x*-axis in walking direction, *y*-axis pointing left and *z*-axis vertical.

To obtain additional information about (rotational) accelerations, which are invariant to the position on the segment, the 3D angular acceleration ***α***^*g*^ was calculated:

(7) $$\alpha^{g}=\frac{d\omega^{g}}{\text{dt}}$$

In the remainder of this paper ***a***, ***ω*** and ***α*** are always expressed in frame *ψ*_*g*_, the index ()^*g*^ is omitted for readability.

### Feature extraction

Features were extracted from magnitudes as well as from the *x*-, *y*-, and *z*-components of the 3D accelerations (***a***), angular velocities (***ω***) and angular accelerations (***α***). The features that were extracted are RMS, variance, correlation coefficients (cc’s) between (the same components of) sensors on different segments, and inter-axis correlation coefficients (of single sensors) and are listed in Table 1.

**Table 1:** Features used for identifying the inertial sensors

		**Feature**	
**Description**	***a***	***ω***	***α***
RMS of the			
-magnitude	RMS{ ||***a***||}	RMS{ ||***ω***||}	RMS{ ||***α***||}
-*x*-component	RMS{ *a*_*x*_}	RMS{ *ω*_*x*_}	RMS{ *α*_*x*_}
-*y*-component	RMS{ *a*_*y*_}	RMS{ *ω*_*y*_}	RMS{ *α*_*y*_}
Variance of the			
-magnitude	Var{ ||***a***||}	Var{ ||***ω***||}	Var{ ||***α***||}
-*x*-component	Var{ *a*_*x*_}	Var{ *ω*_*x*_}	Var{ *α*_*x*_}
-*y*-component	Var{ *a*_*y*_}	Var{ *ω*_*y*_}	Var{ *α*_*y*_}
-*z*-component	Var{ *a*_*z*_}	Var{ *ω*_*z*_}	Var{ *α*_*z*_}
Sum of cc’s of a sensor with all other sensors of the			
-magnitude	*Σ*cc{ ||***a***||}	*Σ*cc{ ||***ω***||}	*Σ*cc{ ||***α***||}
-*x*-component	*Σ*cc{ *a*_*x*_}	*Σ*cc{ *ω*_*x*_}	*Σ*cc{ *α*_*x*_}
-*y*-component	*Σ*cc{ *a*_*y*_}	*Σ*cc{ *ω*_*y*_}	*Σ*cc{ *α*_*y*_}
-*z*-component	*Σ*cc{ *a*_*z*_}	*Σ*cc{ *ω*_*z*_}	*Σ*cc{ *α*_*z*_}
The maximum value of the cc’s of a sensor with all other sensors of the			
-magnitude	Max{cc{ ||***a***||}}	Max{cc{ ||***ω***||}}	Max{cc{ ||***α***||}}
-*x*-component	Max{cc{ *a*_*x*_}}	Max{cc{ *ω*_*x*_}}	Max{cc{ *α*_*x*_}}
-*y*-component	Max{cc{ *a*_*y*_}}	Max{cc{ *ω*_*y*_}}	Max{cc{ *α*_*y*_}}
-*z*-component	Max{cc{ *a*_*z*_}}	Max{cc{ *ω*_*z*_}}	Max{cc{ *α*_*z*_}}
The inter-axis cc’s of a sensor between the			
-*x*- and *y*-axes	cc{ *a*_*x*_,*a*_*y*_}	cc{ *ω*_*x*_,*ω*_*y*_}	cc{ *α*_*x*_,*α*_*y*_}
-*x*- and *z*-axes	cc{ *a*_*x*_,*a*_*z*_}	cc{ *ω*_*x*_,*ω*_*z*_}	cc{ *α*_*x*_,*α*_*z*_}
-*y*- and *z*-axes	cc{ *a*_*y*_,*a*_*z*_}	cc{ *ω*_*y*_,*ω*_*z*_}	cc{ *α*_*y*_,*α*_*z*_}

All 57 (19 ·3) features are given as input to the decision tree learner. The C4.5 algorithm automatically chooses the features that split the data most effectively.

Because the correlation coefficients were in matrix form, they could not be inserted directly as features (because the identity of the other sensors was unknown). For this reason, the sum of the correlation coefficients of a sensor with all other sensors and the maximum value of the correlation coefficients of a sensor with the other sensors were used as features. This corresponds to the sums and the maximum values of each row (neglecting the autocorrelations on the diagonal) of the correlation matrix respectively and gives an impression of the correlation of a sensor with all other sensors. Minimal values and the sum of the absolute values of the correlation coefficients were also investigated, but did not contribute to the identification of the sensors.

### Classification for full-body configurations

Following feature extraction, Weka (Waikato Environment for Knowledge Analysis), a collection of machine learning algorithms for data mining tasks [12,13], was used for the classification of the inertial sensors.

In this study decision trees were used for classification, because they are simple to understand and interpret, they require little data preparation, and they perform well with large datasets in a short time [14,15].

The datasets for classification contained instances of 31 walking trials of 17 sensors each. All 57 features that are listed in Table 1 were given as input to Weka. The features were ranked, using fractional ranking (also known as “1 2.5 2.5 4” ranking: equal numbers receive the mean of what they would receive when using ordinal ranking), to create ordinal features. This was done to minimize variability between individuals and between different walking speeds. This ranking process of categorizing the features is a form of classification and can only be used when the sensor-configuration is known beforehand (in this case it was known that a full-body configuration was used). A drawback of this ranking process is that the distance between the feature values (and thus the physical meaning) is removed.

In Weka, the J4.8 decision tree classifier – which is an implementation of the C4.5 algorithm – with default parameters was chosen. As a test option, a 10-fold cross-validation was chosen because in the literature this has been shown to be a good estimate of the error rate for many problems [15].

The C4.5 algorithm builds decision trees from a set of training data using the concept of information entropy. Information entropy *H* (in bits) is a measure of uncertainty and is defined as:

(8) $$H=-\overset{n}{\underset{i=1}{\sum }}p(i)\underset{2}{\log}(p(i))$$

where *n* is the number of classes (in this case body segments) and *p*(*i*) is the probability that a sensor is assigned to class *i*. This probability is defined as the number of sensors attached to segment *i* divided by the total number of sensors. Information gain is the difference in entropy, before and after selecting one of the features to make a split [15,16].

At each node of the decision tree, the C4.5 algorithm chooses one feature of the dataset that splits the data most effectively, that is, the feature with the highest information gain is chosen to make the split.

The main steps of the C4.5 algorithm are [15,16]: 

1. If all (remaining) instances (sensors) belong to the same class (segment), then finish

2. Calculate the information gain for all features

3. Use the feature with the largest information gain to split the data

4. Repeat steps 1 to 3.

To improve robustness, the classification was split into three steps. In the first step the body segments were classified without looking at left or right (or contra-/ipsilateral), while in the next steps the distinction between left and right was made.

#### *Step one – segment identification*

In the first step, the body segments were identified, without distinguishing left and right. The features were ranked 1-17, but sensors were classified in ten different classes (pelvis, sternum, head, shoulder, upper arm, forearm, hand, upper leg, lower leg and foot), using Weka as described above.

#### *Step two – left and right upper arm and upper leg identification*

When segments were identified in step 1, left and right upper legs (and arms) were identified using correlation coefficients between pelvis-sensor (sternum-sensor for the upper arms) orientation *θ* and upper leg (or arm) movement.

The sternum- and pelvis-sensor orientation *θ* about *x*, *y* and *z* axes were obtained by trapezoidal numerical integration of angular velocity, followed by detrending. In this case it was not necessary to use differential equation (1), because in all directions only small changes in orientation were measured on these segments. This provides left and right information, because of the coordinate frame transformation described before in the preprocessing Section (the *y*-axis points left). For the upper arms and upper legs, accelerations, velocities, angular velocities, angular accelerations and orientations of *x*, *y* and *z* axes were used.

Correlation coefficients of 45 combinations of *x*, *y*, *z* components were calculated, ranked and used to train a decision tree using the same method as described above.

#### *Step three – left and right identification for shoulders, forearms, hands, lower legs and feet*

Left and right identification of the remaining segments (shoulders, forearms, hands, lower legs and feet) was done using correlation coefficients between (*x*, *y*, *z* or magnitude) accelerations and angular velocities of sensors on adjacent segments for which it is known whether they are left or right.

### Classification for lower body plus trunk configurations

The classification for a lower body plus trunk configuration was similar to the full-body configuration, but instead of 17 inertial sensors, only 8 inertial sensors (on pelvis, sternum, right upper leg, left upper leg, right lower legs, left lower leg, right foot and left foot) were used. In the first step the features were now ranked 1-8, but sensors were classified in 5 different classes (pelvis, sternum, upper leg, lower leg, foot). In steps 2 and 3 the distinction between left and right was made again. The decision trees were trained using the 31 trials of the healthy subjects and subsequently tested, using 10-fold cross-validation, on these 31 trials and also on 17 trials of 7 patients after ACL reconstruction.

## Results

### Full-body configurations

The results of the three steps are described individually below.

#### *Step one – segment identification*

**Figure 2:**
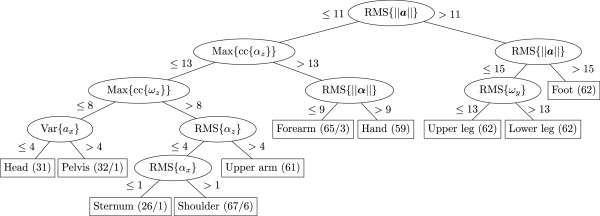
**Decision tree for segment identification (step 1).** Constructed with the J4.8 algorithm of Weka. 31 walking trials of 10 different healthy subjects were used. As testing option a 10-fold cross-validation was used. From the (31·17=)527 inertial sensors, 514 were correctly classified (97.5%). The numbers at the leaves (the rectangles containing the class labels) indicate the number of sensors reaching that leaf and the number of incorrectly classified sensors. For example, 26 sensors reach the sternum leaf, of which one is not a sensor attached to the sternum.

The J4.8 decision tree classifier, as constructed using Weka, is shown in Figure 2. The corresponding confusion matrix is shown in Table 2. From the (31 ·17=) 527 inertial sensors, 514 were correctly classified (97.5%).

**Table 2:** Confusion matrix

**a**	**b**	**c**	**d**	**e**	**f**	**g**	**h**	**i**	**j**	***<*— classified as**	
30	0	1	0	0	0	0	0	0	0	a	Pelvis
0	25	0	6	0	0	0	0	0	0	b	Sternum
1	0	30	0	0	0	0	0	0	0	c	Head
0	1	0	61	1	0	0	0	0	0	d	Shoulder
0	0	0	0	61	0	0	0	0	0	e	Upper arm
0	0	0	0	0	62	0	0	0	0	f	Forearm
0	0	0	0	0	3	59	0	0	0	g	Hand
0	0	0	0	0	0	0	62	0	0	h	Upper leg
0	0	0	0	0	0	0	0	62	0	i	Lower leg
0	0	0	0	0	0	0	0	0	62	j	Foot

Resulting from testing the decision tree in Figure 2 with 10-fold cross-validation, using 31 walking trials. From the (31 ·17=)527 inertial sensors, 514 were correctly classified (97.5%).

The decision making is based on the ranking of the features. For example, when looking at the top of the decision tree (at the first split) the 6 sensors (of each trial) with the largest RMS magnitude of the acceleration (RMS{ ||***a***||}) are separated from the rest. These are the upper legs, lower legs and feet. Consequently the other 11 sensors of each walking trial are the pelvis, sternum, head, shoulders, upper arms, forearms and hand.

#### *Step two – left and right upper arm and upper leg identification*

**Figure 3:**
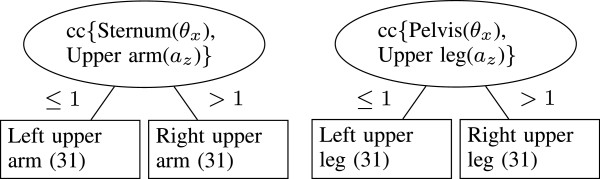
**Decision trees for left and right upper arm and upper leg identification (step 2).** To identify left and right upper arms, from both upper arm sensors the correlation of the acceleration in *z* direction with the sternum sensor orientation about the *x*-axis was used (left). For the upper legs the orientation of the pelvis sensor was used (right). For these segments, all sensors were identified correctly (100% accuracy).

In Figure 3, the decision trees that were constructed for left and right upper arm and upper leg identification are shown. The left Figure indicates that, to identify left and right upper arms, from both upper arm sensors the correlation of the acceleration in *z* direction with the sternum sensor orientation about the *x*-axis has to be calculated. The sensor which results in the largest correlation coefficient is the sensor on the right upper arm. For the upper legs the orientation of the pelvis sensor is used instead of the sternum sensor (right Figure). For these segments, all sensors were identified correctly (100% accuracy).

#### *Step three – left and right identification for shoulders, forearms, hands, lower legs and feet*

Table 3 lists the correlation coefficients for left and right identification of the remaining segments (shoulders, forearms, hands, lower legs and feet), determined using Weka. For example, to identify left and right shoulders, the correlation coefficients of acceleration in *z*-direction between shoulders and upper arms (from which left and right were determined in the previous step) have to be calculated. The largest correlation coefficient then indicates whether segments are on the same lateral side or not. This step also resulted in 100% correct identification.

**Table 3:** Correlation coefficients (cc’s) used for left and right identification in step 3

**Segments**	**cc’s with**	**Component**
Shoulders	Upper arms	*a*_*z*_
Forearms	Upper arms	*a*_*x*_
Hands	Forearms	*a*_*y*_
Lower Legs	Upper legs	*a*_*x*_
Feet	Lower legs	*a*_*x*_

The “cc’s with”-column indicates the segments – for which it is known whether they are left or right – used for determining the component (third column, constructed with J4.8 algorithm in Weka) to determine left and right segments.

### Lower body plus trunk configurations

The results of the three steps are again described individually below.

#### Step one – segment identification

**Figure 4:**
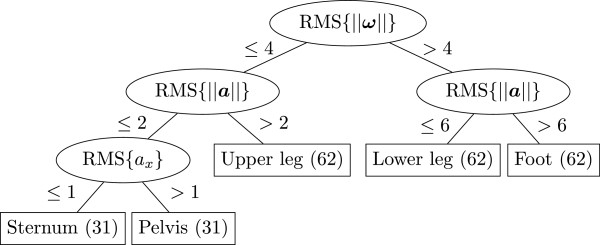
**Decision tree for segment identification (step 1), when using a lower body plus trunk configuration.** 31 walking trials were used (31·8=248 sensors). 10-fold cross-validation was used for testing the tree, resulting in 248 (100%) correctly classified inertial sensors.

The decision tree for lower body plus trunk identification is shown in Figure 4. To train this tree, 31 walking trials were used (31·8=248 sensors). 10-fold cross-validation was used for testing the tree, resulting in 248 (100%) correctly classified inertial sensors.

#### *Step two – left and right upper arm and upper leg identification*

For left and right upper leg identification the tree from Figure 3 can be used again, which resulted in 100% correctly classified sensors.

#### *Step three – left and right identification for remaining segments*

This step is also the same as the left and right leg identification in the full-body configuration case (see Table 3), that is, the correlations of acceleration in *x* direction between upper and lower legs and between lower legs and feet were used, resulting in 100% correctly classified sensors.

### Testing the lower body plus trunk identification algorithms on the patients

The decision trees trained using the walking trials of the healthy subjects were tested on the walking trials of the patients, after the ACL reconstruction. This resulted in 100% correctly identified inertial sensors in all three steps.

## Discussion

The decision trees were trained with features extracted from walking trials involving healthy subjects. It is assumed that the system ‘knows’ the movement of a subject using for example, movement classification algorithms as described in literature [14,17]. This is important, because for our current method the subject needs to be walking. Our expectation is that the identification will become more robust when combining the current classification method with other daily-life activities. For example, when standing up from sitting the sensors on the upper legs rotate approximately 90°, which make these sensors easy to identify. These other activities could then be monitored using activity classification as described, for example, in [14,17], provided that this is possible without having to know the segment to which each sensor is attached beforehand. Then, based on this information, the correct decision tree for identifying the sensors can be chosen. Several new features (such as peak count or peak amplitude) will be needed when other activities are investigated.

It is not always essential (or even desirable) to use a full-body configuration, for example the ACL patients, where the interest is mainly on the gait pattern and the progress in the rehabilitation process. If not all the sensors are used, there are two options. The first option is to use a known subset of the 17 inertial sensors and to use decision trees that are trained using this subset of the sensors. This was shown for a lower body plus trunk configuration, but can be done similarly for every desired configuration, using the same methods. If it is not clear which segments are without sensors, the correlation features between different sensors and the ranking can not be used anymore, because these are both dependent on the number of sensors that is used (if for instance the sensors on the feet are missing – and this is not known – the sensors on the lower legs will be classified as if they are on the feet). A second option that can be used in this case, is to use a new decision tree that was created with features of all the 17 inertial sensors, but without the ranking (so using actual RMS and variance values) and without the correlation coefficients between different sensors (on the other hand, inter-axis correlation coefficient could be used, because they are not dependent on other sensors). To demonstrate this, a decision tree was constructed, which resulted in 400 of 527 correctly classified instances (75.9%). A possible explanation for this decreased performance could be the fact that – because of variations in walking speeds and or arm movements between different walking trials – there is more overlap in the (unranked) features, decreasing the performance of arm and leg identification. This implies that the ranking of the features is a suitable method for reducing the overlap of features between different trials. Another option of minimizing variability between subjects and walking speeds is to normalize the features. We tested this by creating a decision tree with normalized instead of ranked features. This resulted in 461 (87.5%) correctly classified sensors.

To obtain an indication of the sensitivity to changes in feature values, for each feature in the decision tree in Figure 2, the difference between feature-value of each sensor and split-value was calculated. For example, for the feature at the top of the tree, RMS{ ||***a***||}, the 17 RMS values were ranked and the split-value, that is, the mean RMS of ranks 11 and 12 was calculated. Subsequently, the difference between RMS value of each sensor and split-value was calculated (and normalized for each trial), resulting in a measure for the sensitivity to changes in acceleration. If differences are small, even small changes in acceleration can cause incorrectly classified sensors. These differences were calculated for all eight features used in the decision tree and for all trials. For each sensor the mean, variance, minimum and maximum was calculated. From this we concluded that RMS{ ||***a***||}, splitting the sensors on the legs from the other sensors, is not sensitive to changes (in acceleration) and RMS{ ***α***_*x*_}, splitting the sternum- and shoulder-sensors, is very sensitive to changes (in angular acceleration about the *x*-axis), as can also be concluded from the confusion matrix (Table 2) where six sternum-sensors were classified as shoulder-sensors (and one vice versa) and all sensors on the legs were correctly classified.

The measurements used in this study involved placing the inertial sensors on the ideal positions as described in the Xsens MVN user manual to reduce soft tissue artifacts [9]. But what is the influence of the sensor positions on the accuracy of the decision tree? Will the sensors be classified correctly if they are located at different positions? To answer this question a decision tree without the translational acceleration features was investigated, because on a rigid body the angular velocities (so also the angular accelerations) are considered to be the same everywhere on that rigid body. This tree for segment identification resulted in an accuracy of 97.2% (512 of 527 sensors correctly classified). The tree without the translational accelerations also introduced errors in the left and right identification, for example, the left and right upper arm and upper leg identification both resulted in 60/62 (96.8%) correctly classified sensors. To gain a better understanding of the influence of the sensor positions, additional measurements are required.

In current motion capture systems, data from several inertial sensors is collected and fused on a PC running an application that calculates segment kinematics and joint angles. This application currently requires information about the position of each sensor, which is handled by labeling each sensor and let the user attach it to the corresponding body segment. The algorithm presented in this paper can be implemented in this application and take over the responsibility of the correct attachment from the user, with the additional advantage to reduce possible attachment errors. Consequently, the procedure must guarantee a 100% correct identification, which will not always be the case. Therefore, a solution for this problem could be for the user to perform a visual check via an avatar – representing the subject that is measured – in the running application. If the movement of the avatar does not correspond to the movement of the subject, the subject is asked to walk a few steps to which the identification algorithm can be applied again. In addition to this, the system detects the activity the subject performs and can hence apply the algorithm several times during a measurement and alarm the user if the classifications do not fully correspond.

In this study, a decision tree classifier was used resulting in 97.5% correctly classified sensors. Other classifiers were investigated. For example a support vector machine (SVM) as used by Amini *et al.* in [8] resulted in 518/527 (98.3%) correctly classified sensors when a radial basis function was used with best parameters obtained using cross-validation (“CVParameterSelection” in Weka) [15]. Disadvantage, however, is that the resulting parameters of the hyperplanes are not as easy to interpret as decision trees.

Other differences with previous studies, as described in the Introduction, are the number of sensors used. While in [6,8] respectively 5 and 10 inertial sensors were used, our algorithm provides identification for full-body configurations (17 inertial sensors). Whereas in these previous studies only acceleration features (in sensor coordinates) were used, we also use angular velocities – reducing the influence of the position of the sensor on the segment – and rotated sensor data to a global coordinate frame, for a 3D comparison of movement data from different subjects and allowing left and right identification.

Currently the results are based on three walking cycles. Increasing the trial length (which was possible for most of the recorded trials) did not improve accuracy, whereas a decrease resulted in accuracies of 92.6% when using two walking cycles and 90.1% when using one walking cycle (without looking at left and right identification). When using one and a half walking cycle, the accuracy was 92.0%, hence using multiples of full walking cycles seems no necessity.

To test the influence of integration drift on the estimation of the walking direction, we added an error angle to the angle *θ* from (5). The accelerometer bias stability is 0.02 m/s^2^[2], which can cause a maximum error in velocity of 0.06 m/s after integrating over three seconds (the first walking cycle was always within three seconds). This subsequently leads to an error in the angle *θ* of 3.5 degrees. We added a random error angle, obtained from a normal distribution with standard deviation of 3.5 degrees to the angle *θ*. From this we calculated the features and tested them on the decision trees constructed using the normal features. This resulted in 97.7% correctly classified sensors in step one and 100% correctly classified sensors in the steps two and three. For an error angle of 10 degrees 97.2% of the sensors were correctly classified in step one. In steps two and three all sensors were correctly classified, except for the upper legs, from which 96.8% of the sensors were correctly classified.

No outstanding differences between male and female subjects were observed.

## Conclusions

A method for the automatic identification of inertial sensor placement on human body segments has been presented. By comparing 10 easy to extract features, the body segment to which each inertial sensor is attached can be identified with an accuracy of 100.0% for lower body plus trunk configurations and 97.5% for full-body configurations, under the following constraints, which are satisfied in most practical situations: 

•From a standing start (so the initial sensor inclination in the global frame can be obtained) the subject starts walking normally in a straight line, with sufficient arm movement.

•The sensor configuration needs to be known.

The features were extracted from magnitudes and 3D components of accelerations, angular velocities and angular accelerations, after transforming all signals to a global coordinate frame with *x*-axis in walking direction, *y*-axis pointing left and *z*-axis vertical. Identification of left and right limbs was realized using correlations with sternum orientation for upper arms and pelvis orientation for upper legs and for remaining segments by correlations with sensors on adjacent segments. We demonstrated the robustness of the classification method for walking in ACL reconstruction patients.

When the sensor configuration is unknown, the ranking and the correlation coefficients between sensors cannot be used anymore. In this case, only 75.9% of the sensors are identified correctly (that is 400 of 527 sensors, based on a full body configuration). If it is known which sensors are missing, another decision tree without the missing sensors can be used. If the sensors are not attached to the optimal body positions, decision trees which only use features extracted from angular velocities and angular accelerations can be used instead.

## Competing interests

The authors declare that they have no competing interests.

## Authors’ contributions

DW developed and tested the sensor to segment identification algorithm and drafted the manuscript. BB, CB and PV assisted with data interpretation and helped to develop the algorithm. BB and PV helped to draft the manuscript. BB, CB, HH and PV supervised the research. CB provided the data of the ACL patients and revised the manuscript. All authors read and approved the final manuscript.
